# Efficacy of Manuka honey oral rinse in treatment of xerostomia among elderly patients: a randomized controlled trial

**DOI:** 10.1186/s12903-025-06125-9

**Published:** 2025-05-23

**Authors:** Dalia Ghalwash, Ayman El-Gawish, Asmaa Abou-Bakr

**Affiliations:** 1 Oral Medicine and Periodontology Department, Faculty of Dentistry, The British University in Egypt, El Sherouk City, Egypt; 2 Oral Medicine and Periodontology, Faculty of Dentistry, Galala University, Suez, Egypt

**Keywords:** Manuka honey, Antioxidant, Xerostomia, Treatment, Elderly

## Abstract

**Background:**

Xerostomia is a common complaint among elderly patients. Due to the anti-inflammatory effect, Manuka honey could be a promising alternative remedy for many medical conditions, including xerostomia. The present study aimed to evaluate the effectiveness of Manuka honey oral rinse as a novel management for xerostomia in elderly patients.

**Methods:**

This study was designed as a randomized, single-blinded clinical trial. 42 elderly patients who were all evaluated for the presence of xerostomia and hyposalivation were randomly allocated into 3 equal groups, the interventions were used 3 times per day for 1 month as follows: Manuka honey oral rinse in group I, natural honey oral rinse in group II, and saline in group III (control). The Summated Xerostomia Inventory (SXI) score, The Clinical Oral Dryness Score (CODS), and the salivary flow rate were evaluated for all groups at different intervals. The Oral Health Impact Profile (OHIP-14) questionnaire was assessed 1 month after intervention.

**Results:**

Manuka honey oral rinse caused a significant reduction of the subjective SXI score (2 ± 0.39) and objective clinical oral dryness (5.71 ± 0.91) scores compared to the other 2 groups. Moreover, the salivary flow rate was significantly higher after one month of using Manuka honey oral rinse (1.51 ± 0.34) than honey oral rinse group (1.01^a^ ± 0.19), and the control group (0.81a ± 0.19). The most significant improvement of OHIP scores was seen in the Manuka honey group (5.50 ± 1.16) compared to the honey group (7.57^a^ ± 1.02), and the control group (9.36^ab^ ± 1.78).

**Conclusion:**

Manuka honey oral rinse demonstrated high efficiency in the management of xerostomia among elderly patients when compared with natural honey as it relieved the symptoms and severity of xerostomia in the elderly along with a high rate of patient satisfaction.

**Trial registration:**

The study was registered at Clinical Trials.gov (NCT06240806) on 01/14/2024.

## Background

Aging is an unavoidable biological process and is largely viewed as the deteriorating function of most body organs due to the progressive accumulation of tissue damage [1]. It is expected that the global aging population above the age of 60 years will almost double to 1.5 billion by 2050 [2]. This will deepen the worldwide burden of oral disorders in older adults [3].

Xerostomia is the subjective sensation of dryness of the mouth, while hyposalivation is an objective reduced salivary flow rate both are very common in the aging population [4]. Xerostomia and hyposalivation are common oral dysfunctions that might be attributed to aging, extensive medications, and various local and systemic factors [5–7].

Owing to the important functions and biological benefits of saliva, xerostomia patients with or without hyposalivation may experience complications such as halitosis, dysgeusia, periodontitis, oral discomfort, increased caries rate, candidiasis, burning sensation, dysphagia, and impaired immunological responses leading to poor overall quality of life [8–10].

Management options for xerostomia include using salivary substitutes in cases of total salivary gland damage and using salivary stimulants in cases of remaining salivary gland function [11]. However, there is a lack of robust evidence supporting the use of one treatment over the other and the results are mostly not entirely satisfactory. Hence, searching for unconventional therapeutic options to treat xerostomia is still a high necessity.

Several natural agents demonstrate promising pharmacologic effects that could successfully alleviate dry mouth. They can enhance salivary gland function through molecular pathways, including modulation of antioxidant enzymes, suppression of autoantigens, and affecting cell proliferation [12, 13]. Furthermore, certain natural products have a moisturizing effect on oral mucosa, possibly improving xerostomia. Moreover, selected natural agents have antioxidant and anti-inflammatory properties that enhance the gustatory system function and hydration of the oral mucosa constituting a promising remedy for xerostomia [14, 15].

Oxidative stress occurs when the reactive oxygen species (ROS) level exceeds the antioxidant level. As age increases, accumulation of more ROS happens leading to oxidative damage to proteins, lipids, and DNA. Our bodies are armed with a variety of antioxidant molecules to guard them against the damaging effect of ROS buildup, which could be further augmented by antioxidant supplements [16, 17].

One powerful natural antioxidant with countless biological properties is Manuka honey. Manuka honey is a mono-floral honey that is well recognized for its beneficial values which are mainly ascribed to its distinctive chemical structure as it stands apart from any other types of honey due to its high amount of flavonoids, particularly luteolin (> 0.05 mg/100 g honey) [18]. It is full of macro-and micro-nutrients including, proteins, enzymes, sugars, minerals, amino acids, and vitamins [19]. These valuable phytochemicals are directly related to their special medical benefits including wound healing, antimicrobial, antioxidant, anticancer, and anti-inflammatory properties [20].

Honey, the natural remedy with its rich and complex constituents and great nutritional value, has variable health-related benefits and pharmaceutical applications without causing negative side effects like other therapeutic agents [21]. Manuka honey has a higher content of polyphenolic compounds compared with other classes of honey; phenolic acids, flavonoids, methoxylated benzoic acid, and syringic acid, besides non-phenolics as vitamin E, vitamin C, and β-carotene. Thus, it has great anti-inflammatory and antioxidant properties [22, 23]. The main source of Manuka honey’s unique antibacterial qualities as well as a number of other health advantages is methylglyoxal (MGO), which is created during the honey’s maturation and aging process. Because of its unique methylglyoxal (MGO) content, MH, for example, is widely prized for its non-peroxide antibacterial qualities [20].

The antioxidant action of Manuka honey is evident in immune cells such as neutrophils and macrophages, it was also reported to guard human fibroblasts against oxidative stress probably owing to the high level of methyl syringate and leptosin byproducts [19]. Studies have revealed that manuka honey can impede the respiratory burst of neutrophils and ROS formation, recover oxidative stress, and enhance enzymatic (glutathione peroxidase, catalase, and superoxide dismutase) and non-enzymatic (glutathione) actions [24]. Manuka honey is also a powerful anti-inflammatory agent due to its phenolic compounds mainly quercetin and gallic acid as they can increase cell viability via downregulation of caspase 3, and enhance wound healing [25].

Moreover, manuka honey was found to possess a hydrating and moistening effect probably due to the high moisture contents of MH which constitutes around 17–20% of its total weight, which is why it has been used in moisturizing cream products [26]. Manuka honey was recently used in several studies in the form of eye drops and it revealed significant advantages and potential for managing the subjective symptoms of dry eye [24]. The authors supposed that the hypertonicity of manuka honey caused the attraction of liquids from deeper tissues to the ocular surface forming a lubricating layer to preserve the surface moist [24]. This reason might also apply to the moistening effect of manuka honey on the mucosal surfaces of the oral cavity hence improving the patient’s symptoms of xerostomia.

These significant and variable biological functions, particularly the potent antioxidant activity, which could protect the body from oxidative damage caused by aging, in addition to the anti-inflammatory effect propose that Manuka honey could be a promising alternative remedy in plentiful medical conditions including xerostomia. Therefore, we hypnotized that using Manuka honey as an oral rinse could improve both subjective and objective scores of dry mouth as well as increase the salivary flow rates.

## Methods

### Study design and aim

This study was designed as a single-center randomized controlled comparative, single-blinded clinical trial. The present investigation aimed to evaluate the clinical effectiveness of Manuka honey oral rinse in the management of xerostomia in the elderly through assessment of subjective and objective dry mouth, along with assessment of salivary flow rate, and the impact on their quality of life.

### Sample size calculation and grouping

To apply a statistical test of the null hypothesis which states that there is no difference between the tested groups regarding the perception of xerostomia a power analysis was created with sufficient power. After utilizing alpha (α) and beta (β) levels of (0.2) (i.e., power = 95%) and computing a critical z-value of (1.96), which was derived from the outcomes of an earlier investigation by *Ibrahim et al.* [27],, the smallest necessary sample size (n) was determined to be14 cases for each group. G*Power 3.1.9.7 was used to calculate the sample size.

### Randomization and blinding

Participants were assigned in blocks of four to one of the two study groups by the use of computer-generated random allocation software (Simple Randomization Service| Sealed Envelope [Internet]) and the permuted block technique. Each allocation was enclosed in opaque envelopes and provided with a code. A trial-independent person carried out the randomization sequence; this person was also in charge of maintaining the envelopes and only unfolding them when the intervention occurred. Every patient’s type of treatment was concealed. The outcome assessor was blinded and unaware of which kind of treatment was given to the patients.

### Patient selection

All participants were elderly patients complaining of xerostomia, selected from the diagnostic center at The British University of Egypt. The following question, reported in prior studies [28, 29], was asked to them before participating in the study to validate their experience of dry mouth and xerostomia: “How often do you feel that your mouth is dry?” The options that participants may choose from were “never,” “sometimes,” “usually,” or “always.” Individuals who responded with “usually” or “always” were deemed to have xerostomia and were incorporated into the present investigation [29].

Additionally, to validate the hyposalivation along with dry mouth, the salivary flow rates were also measured. A sialometry test is the clinical technique most frequently used to diagnose salivary malfunction. When the unstimulated salivary flow rates are less than 0.1 mL/min, hyposalivation is proven. The salivary flow rate in the unstimulated range of 0.3 to 0.4 ml/min [30].

The British University in Egypt Research Ethics Committee (REC 23–061) granted ethical approval for the study, which was then filed at https://clinicaltrials.gov with the NCT06240806 number. Patients completed an informed consent form after receiving a thorough explanation of the operations. The study was performed as per the principles of the modified Helsinki’s code for human clinical studies (Association, 2013), and CONSORT 2010 guidelines for reporting randomized clinical trials.

Then after selecting the elderly patients with xerostomia along with hyposalivation, they underwent another screening using the following standards prior to recruitment to the 3 different groups:

### Inclusion criteria

- Adults over 65, regardless of gender.
- Individuals who only responded with “usually” or “always” when they were asked, “How often do you feel that your mouth is dry?” [28, 29].
- The unstimulated salivary flow rates are less than 0.1 mL/min [30].
- Patients with well-controlled diabetes mellitus or hypertension using their prescribed medications regularly.
- Individuals with xerostomia for at least three months before participation [31].
- Patients were able to make reliable decisions or communications.

### Exclusion criteria

- Smoking and alcohol consumption.
- Patients with a history of malignancy or head and neck radiotherapy.
- Patients with any current oral mucosal lesions.
- Individuals previously received treatment for autoimmune conditions such as lupus, rheumatism, or Sjögren’s syndrome [31].
- Patients who used any treatments or drugs to alleviate xerostomia [31].
- Handicapped patients, severely disabled, mentally disabled individuals, or patients who refused to participate [32].

**Fig. 1:**
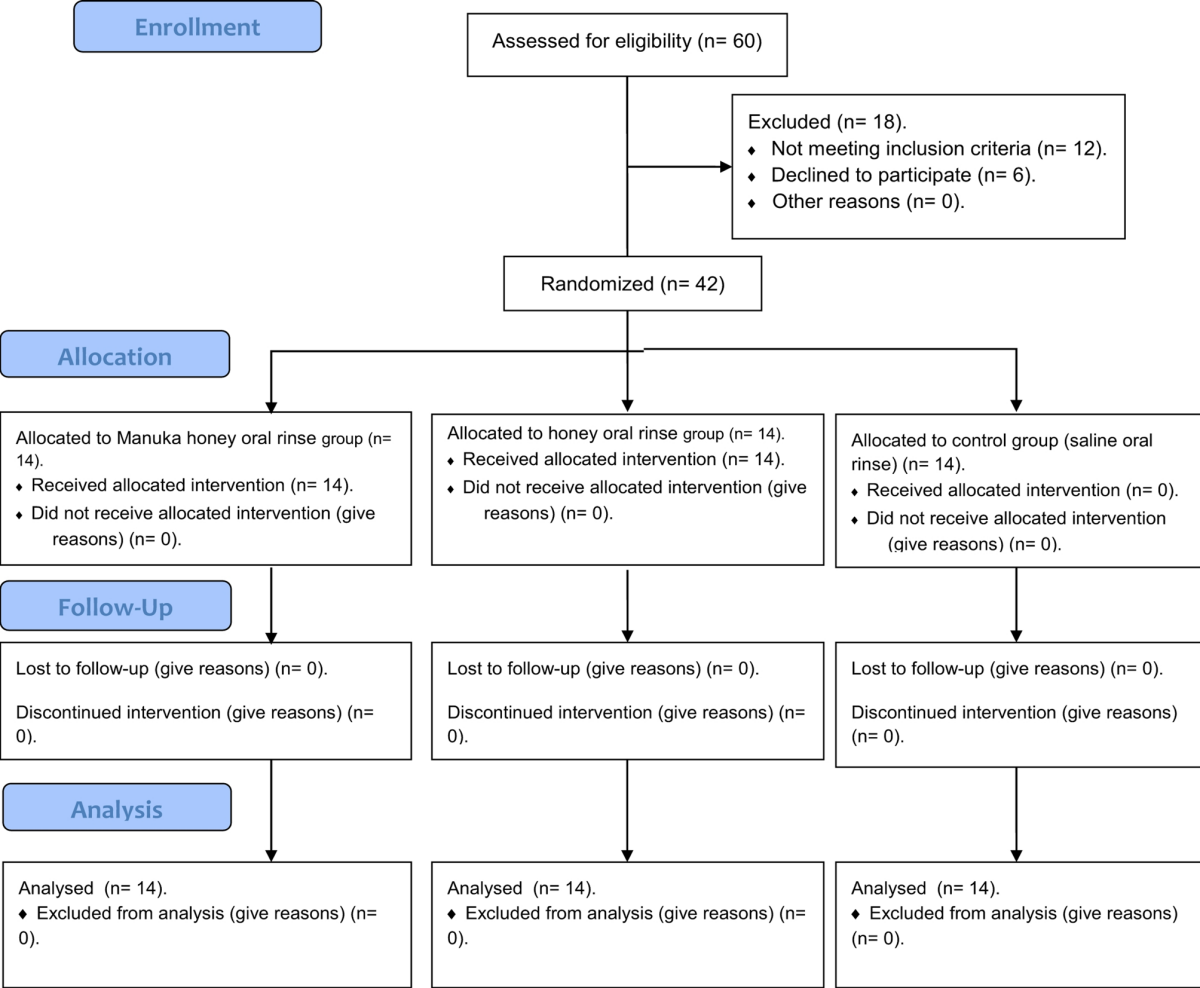
Flow chart for patient’s recruitment

All patients who met all the eligibility criteria were then asked about the history of their underlying systemic condition, and the list of regular medications used. They were also subjected to clinical examination to assess the general condition of the oral cavity and detect any oral mucosal lesions. The flow chart for patient recruitment is illustrated in Fig. 1.

### Treatment protocol

#### Group I (Manuka honey oral rinse)

- New Zealand Manuka honey used in this study was purchased from Amazon Egypt.
- As per the administration protocol of Ibrahim et al. [27], manuka honey was utilized as an oral rinse. The oral rinses, which were made by the principal investigator A.A (20 ml of manuka honey diluted in 100 ml of filtered water) and given to the patients in 250 ml plastic bottles (four for each patient), were used by the patients three times a day for 30 days.

#### Group II (natural honey oral rinse)

- **Honey** (100% Natural) was purchased from Amazon Egypt.
- It was prepared as 20 ml of honey diluted in 100 ml of filtered water and used 3 times per day for 30 days.

#### Group III (Saline oral rinse)

- Patients in the control group rinsed using saline as usual, according to the same regimen 3 times per day for one month.

Since natural honey and Manuka honey oral rinses are both different in color, as well as in taste from saline oral rinse, therefore the patients were not blinded to the type of treatment they received.

Additionally, all patients were instructed to recognize and report any adverse reactions that might occur due to Manuka honey.

### Evaluation of outcomes for all groups

At baseline, two weeks, and one month following the initiation of the treatment procedure, the SXI score, CODS score, salivary flow rate, and OHIP-14 were assessed.

The following criteria were used to evaluate the treatment assessment:

#### The primary outcome

##### The summated Xerostomia inventory (SXI) [33, 34]

Using the SXI score, which asks patients to rate the frequency of dry mouth complaints about five statements collected at three distinct periods (baseline, after two weeks, and after one month). The SXI has a maximum score of 5, which denotes serious issues with dry mouth [34].

1. After a meal, my mouth feels dry.
2. I have a dry mouth.
3. I find it challenging to eat dry meals.
4. I have trouble swallowing some food.
5. My lips look chapped.

#### The secondary outcomes

##### The clinical oral dryness score (CODS) [34–36]

Based on several indicators of oral dryness, the CODS is intended to evaluate oral dryness by clinical and visual examination of the oral cavity. It was evaluated at 3 different time intervals (baseline, 2 weeks, one month). A total CODS was calculated by adding the scores from the ten features. Increased severe oral dryness is indicated by a high overall score [36].

1. The mirror adheres to the buccal mucosa.
2. The mirror adheres to the tongue.
3. Slobbering saliva.
4. There isn’t a saliva pool on the tongue.
5. The tongue lacks papillae.
6. Modified or refined gingival architecture.
7. The palate and other oral mucosa appear glassy.
8. Lobulated or fissured tongue.
9. Recent or active (2 teeth).
10. Junk on the palate (not the area beneath the dentures).

- **Salivary flow rate**: In order to reduce the impact of the diurnal variations in salivary composition, samples from patients (during a dialysis visit) were taken between 8:00 AM and 11:00 AM. Before meals, samples were gathered, and speaking was not allowed at that time. Using the spitting method, unstimulated entire saliva was collected for five minutes. To quantify the flow rate (mL/min), the collection was timed [37], 0.3–0.4 mL/min is the average unstimulated salivary flow rate.
- **The OHIP-14**: The OHIP-14 utilizes a scale with five categories (1 = never, 2 = hardly ever, 3 = occasionally, 4 = fairly often, and 5 = very often). A lower score in any of the five categories indicates higher satisfaction [38].

### Statistical analysis of the data

Data was fed to the computer and analyzed using IBM SPSS software package version 20.0. (Armonk, NY: IBM Corp). Categorical data were represented as numbers and percentages. The chi-square test was applied to compare between two groups. Alternatively, the Monte Carlo correction test was applied when more than 20% of the cells had an expected count of less than 5. For continuous data, they were tested for normality by the *Shapiro-Wilk test*. Quantitative data were expressed as range (minimum and maximum), mean, standard deviation, and median for normally distributed quantitative variables Student t-test was used to compare two groups while ANOVA with repeated measures was used to compare between more than two periods and Post Hoc test (Bonferroni adjusted) for pairwise comparisons. The significance of the obtained results was judged at the 5% level.

## Results

The study was conducted on 42 elderly patients randomly assigned to each of the three groups. Table 1 displayed intergroup comparisons and summary statistics for the demographic data, indicating that there was no significant difference in age (*p* = 0.743) or gender (*p* = 0.906), nor the different underlying medical conditions (*p* = 0.586) between the three groups.

### Salivary parameters

#### The summated Xerostomia inventory (SXI)

##### Intergroup comparisons

There was no significant difference between the three groups at baseline or after two weeks (*p* = 0.476 and 0.305, respectively). After one month, the mean values of SXI in the Manuka honey group were significantly reduced compared to both the control and natural honey groups (p = < 0.001*) as shown in Table 2.

#### The clinical oral dryness score (CODS)

##### Intergroup comparisons

After one month the mean CODS scores were lower in the Manuka honey group than the other groups but with no significant difference (*p* = 0.104 respectively) as shown in Table 2.

#### Salivary flow rate

##### Intergroup comparisons

There was a significant difference between the three groups after two weeks and after one month (*p* ≤ 0.05). The Manuka honey group recorded higher salivary flow rates than the other 2 groups with a statistically significant difference, both at 2 weeks and one-month intervals (*p* ≤ 0.05) as presented in Table 3.

#### OHIP score

The intergroup comparison for OHIP scores was shown in Table 3, where the Manuka honey group scores were considerably lower than the other 2 groups (p = < 0.001^*^).

## Discussion

The Aging population is rapidly growing and improving or preserving the health and quality of life of these individuals is challenging due to the intricacy of oral health conditions, abundant systemic disorders, and multiple drug use [39, 40].

As regards the different medical conditions of the participating elderly patients, the current study showed that all patients in all three groups had either diabetes mellitus, hypertension, or a history of COVID-19 with no significant difference between the three groups.

Diabetic patients frequently have xerostomia and hyposalivation. Aging, head and neck radiation, systemic illnesses, and certain medications are among the factors that might cause salivary abnormalities in persons with diabetes mellitus [41, 42]. In addition, there can be dehydration, disruptions in glycemic control, injury to the gland parenchyma, and changes in the microcirculation of the salivary glands [41].

By lowering the number of acinar cells and encouraging fatty infiltration and constriction of the arteries, hypertension may be a factor in the degeneration of the submandibular glands. It has been demonstrated that hypertension affects both the pH and the flow rate of saliva [43]. Moreover, patients with hypertension on anti-hypertensive therapy had a higher prevalence of hyposalivation [44].

The past COVID-19 pandemic has created many obstacles for the medical field until now [45]. Many COVID-19 survivors report having xerostomia even seven months to a year after the illness has passed. Pathogenically, co-occurring gustatory and salivary sequelae are linked to either or both SARS expression receptors important for CoV-2 cellular entrance in salivary glands, and SARS- Zinc shortage brought on by a CoV-2 infection, which is necessary to preserve normal saliva production. Even though COVID-19 oral symptoms are not life-threatening, their prolonged occurrence significantly affects the general quality of life [46, 47].

Aging and age-related diseases are essentially caused by oxidative stress. Therefore, it is considered that oxidative stress is an important cause of salivary gland damage, impaired secretory function, and altered salivary composition in the elderly [48, 49]. Using natural remedies for medicinal purposes has various pros over synthetic medicines. They are more promptly accessible and less costly, adding up, natural products have a highly variable structure, making them an exceptional source of novel therapeutic alternatives [13].

Manuka honey is a powerful antioxidant which is a useful property in the management of xerostomia because oxidative stress is involved in the underlying tissue damage [49]. Additionally, the low cost, availability, lack of preservatives, sterility, long shelf-life, non-toxicity, and insignificant side effects with long-standing administration [25, 50], make manuka honey a promising candidate to be explored as a novel therapeutic alternative for xerostomia management.

Numerous researches were performed to estimate the effectiveness of honey in the management of xerostomia [21, 51–53]. Even though many studies have shown the benefits of Manuka honey against oral diseases such as caries [54], reducing dental plaque [55], and gingivitis [56]. However, no study has ever investigated the effect of manuka honey in the management of xerostomia. Thus, we conducted the first investigation to assess the clinical efficacy of Manuka honey oral rinse in the management of hyposalivation and xerostomia.

In the present study, patients treated with Manuka honey oral rinse had significantly reduced SXI scores than the other 2 groups after one month of therapy (*p* < 0.001), pointing out a diminished feeling of xerostomia. Moreover, reduced objective CODS scores were also encountered in the Manuka honey group after 1 month, reflecting increased salivary flow and this was more validated by the significant rise of salivary flow rate assessed after 1 month in the Manuka honey group which outperformed the natural honey and saline in terms of salivary flow rate enhancement despite the initially reduced salivary flow rate values in Manuka honey group than natural honey and control groups at baseline.

These superior results of Manuka honey compared to natural honey could be attributed to the higher content of polyphenols which enhances its bioavailability and the relatively higher antioxidant capacity of Manuka honey in comparison to other honeys as reported in recent research [23].

Our results are in accordance with a recent study performed to evaluate the efficiency of thyme honey in managing xerostomia in end-stage renal disease geriatric patients as the authors reported that both the objective and subjective dry mouth scores were substantially improved after a month of thyme honey treatment. Additionally, a significant increase in salivary flow rate was reported one month after thyme honey treatment [27].

However, another study examined the alterations in salivary flow rate following tualang honey intake and reported a significant rise in the salivary flow rate after two weeks of treatment [51].

Oral Health Impact Profile (OHIP-14) questionnaire has been used in the present study to assess the impact of manuka honey intervention on the overall well-being of individuals [38], and it showed very satisfactory results as OHIP scores were significantly lower in the intervention group than the control group (*P* > 0.001), denoting the great impact of manuka honey oral rinse on quality of life and the higher patient’s satisfaction.

These successful results in the management of xerostomia particularly in patient-related outcomes are attributed to the diverse biological properties and unique components of manuka honey, most importantly it comprises some organic acids that could enhance salivary flow and excite chemoreceptors in the oral cavity as ascorbic, caffeic and ferulic acid [17]. Ascorbic acids are known for their salivary stimulating effect, and they are used for the management of xerostomia [11]. Caffeic acid is a precursor of ferulic acid which has remarkable antioxidant and anti-inflammatory properties that are mainly due to its phenolic nucleus and the conjugated 3 C-side thus it is reported to exert a variety of health benefits through its antioxidant properties, especially against chronic diseases associated with oxidative damage [57]. Furthermore, luteolin is one of the most common flavones with antioxidant activity and it has been evaluated as being a unique component in Manuka honey [58].

Additionally, Manuka honey contains gallic acid which has been reported to preserve salivary function after radiotherapy by promoting DNA repair and suppressing radiation cytotoxicity in salivary gland cells [59]. Moreover, epigallocatechin was detected in Manuka honey which exhibits a potent anti-antioxidant, inflammatory, and analgesic effect by lowering levels of plasma prostaglandin E2, TNF-α, IL-1β, and IL-6, and it has been found to improve salivary dysfunction [49].

Apigenin, a single active component of manuka honey was reported in recent research to be able to restore salivary flow rates in OVX mice through robustly upregulating the expression of Aquaporin5, a transmembrane channel protein that mediates transcellular water permeability thus playing an important role in salivary secretion with a great potential to treat xerostomia [60].

Similarly, another important flavonoid in manuka honey, quercetin was investigated for its effects on salivary secretion and was found to be effective in treating impaired salivary secretion through augmenting aquaporin 5 expression and calcium uptake and suppressing oxidative stress and inflammation-related tissue damage [61].

Among the limitations of this study are the narrow time interval and limited sample size. Hence, we recommend further studies to validate our results, preferably multi-centered studies with a larger sample size and multiple time intervals with longer duration. Moreover, this study focused on one cause of xerostomia which is age-related damage of salivary gland tissue in elderly patients, thus additional research directed at patients with other causes of xerostomia is advised to estimate the effectiveness of manuka honey in different patients’ categories and populations.

## Conclusions

The therapeutic use of manuka honey oral rinse for one month has significantly reduced the subjective SXI score and objective clinical oral dryness score, along with a significant increase in the salivary flow rates compared to controls thus relieving symptoms and severity of xerostomia in the elderly without known negative side effects. In addition, the significant improvement of OHIP scores in the manuka honey group conveyed the great impact of manuka honey oral rinse on the treated patient’s quality of life with higher patient satisfaction. Manuka honey oral rinse demonstrated high efficiency in managing xerostomia constituting a novel and favorable alternative to the currently used medications.

**Table 1:** Comparison between the three studied groups according to different parameters

	Manuka honey (*n* = 14)	Honey (*n* = 14)	Control (*n* = 14)	Test of Sig.	*p*
**Gender**					
Male	5 (35.7%)	6 (42.9%)	6 (42.9%)	χ^2^=0.198	0.906
Female	9 (64.3%)	8 (57.1%)	8 (57.1%)		
**Age (years)**					
Mean ± SD.	67.7 ± 1.38	67.5 ± 2.18	67.2 ± 1.48	F = 0.300	0.743
Median (Min. – Max.)	67.5 (66–70)	67.5 (64–71)	67 (66–70)		
**Medical condition**					
Diabetes	4 (28.6%)	2 (14.3%)	6 (42.9%)	χ^2^=3.092	^MC^*p*=0.586
Hypertension	5 (35.7%)	7 (50%)	5 (35.7%)		
Post-Covid 19	5 (35.7%)	5 (35.7%)	3 (21.4%)		

SD: Standard deviation χ^2^: Chi square test MC: Monte Carlo

F: F for One way ANOVA test, pairwise comparison bet. each 2 groups were done using Post Hoc Test (Tukey)

p: p value for comparing between the three studied groups

*: Statistically significant at *p* ≤ 0.05

a: Significant with Manuka

b: Significant with Honey

**Table 2:** Comparison between the three studied groups according to SXI and CODS scores

		Manuka honey (*n* = 14)	Honey (*n* = 14)	Control (*n* = 14)	F	*p*
**SXI**	**Baseline**					
	Mean ± SD.	4.07 ± 0.62	3.79 ± 0.70	3.79 ± 0.80	0.756	0.476
	Median (Min. – Max.)	4 (3–5)	4 (3–5)	4 (3–5)		
	**2 Weeks**					
	Mean ± SD.	3.14 ± 0.36	3.36 ± 0.74	3.50 ± 0.65	1.223	0.305
	Median (Min. – Max.)	3 (3–4)	3 (2–5)	3 (3–5)		
	**1 Month**					
	Mean ± SD.	2 ± 0.39	2.64^a^ ± 0.74	3.07^a^ ± 0.47	13.076^*^	< 0.001^*^
	Median (Min. – Max.)	2 (1–3)	2.50 (2–4)	3 (2–4)		
**CODS**	**Baseline**					
	Mean ± SD.	7.43 ± 1.09	7.07 ± 0.92	7.07 ± 0.92	0.623	0.542
	Median (Min. – Max.)	7 (6–9)	7 (6–9)	7 (6–9)		
	**2 Weeks**					
	Mean ± SD.	6.57 ± 1.02	6.64 ± 1.22	6.57 ± 1.09	0.019	0.981
	Median (Min. – Max.)	6.50 (5–8)	7 (5–9)	7 (4–8)		
	**1 Month**					
	Mean ± SD.	5.71 ± 0.91	5.93 ± 1.38	6.57 ± 0.85	2.403	0.104
	Median (Min. – Max.)	6 (4–7)	6 (4–9)	7 (5–8)		

SD: Standard deviation χ^2^: Chi square test MC: Monte Carlo

F: F for One way ANOVA test, pairwise comparison bet. each 2 groups were done using Post Hoc Test (Tukey)

p: p value for comparing between the three studied groups

*: Statistically significant at *p* ≤ 0.05

a: Significant with Manuka

b: Significant with Honey

**Table 3:** Comparison between the three studied groups according to the salivary flow rate and OHIP score

		Manuka honey (*n* = 14)	Honey (*n* = 14)	Control (*n* = 14)	F	*p*
**Flow rate**	**Baseline**					
	Mean ± SD.	0.46 ± 0.23	0.76^ab^ ± 0.26	0.55 ± 0.14	6.722^*^	0.003^*^
	Median (Min. – Max.)	0.45 (0.10–0.90)	0.80 (0.10–1)	0.55 (0.30–0.80)		
	**2 Weeks**					
	Mean ± SD.	0.94 ± 0.33	0.87^b^ ± 0.19	0.61^a^ ± 0.18	6.968^*^	0.003^*^
	Median (Min. – Max.)	1 (0.40–1.50)	0.95 (0.50–1.10)	0.60 (0.30–1)		
	**1 Month**					
	Mean ± SD.	1.51 ± 0.34	1.01^a^ ± 0.19	0.81^a^ ± 0.19	29.530^*^	< 0.001^*^
	Median (Min. – Max.)	1.50 (1–2)	1 (0.70–1.30)	0.80 (0.50–1.20)		
**OHIP**	**1 Month**					
	Mean ± SD.	5.50 ± 1.16	7.57^a^ ± 1.02	9.36^ab^ ± 1.78	28.201^*^	< 0.001^*^
	Median (Min. – Max.)	5 (4–7)	7.50 (6–9)	9.50 (7–12)		

SD: Standard deviation χ^2^: Chi square test MC: Monte Carlo

F: F for One way ANOVA test, pairwise comparison bet. each 2 groups were done using Post Hoc Test (Tukey)

p: p value for comparing between the three studied groups

*: Statistically significant at *p* ≤ 0.05

a: Significant with Manuka

b: Significant with Honey

## Data Availability

No datasets were generated or analysed during the current study.
